# GBshape: a genome browser database for DNA shape annotations

**DOI:** 10.1093/nar/gku977

**Published:** 2014-10-17

**Authors:** Tsu-Pei Chiu, Lin Yang, Tianyin Zhou, Bradley J. Main, Stephen C.J. Parker, Sergey V. Nuzhdin, Thomas D. Tullius, Remo Rohs

**Affiliations:** 1 Molecular and Computational Biology Program, Department of Biological Sciences, University of Southern California, Los Angeles, CA 90089, USA; 2 Departments of Computational Medicine and Bioinformatics and Human Genetics, University of Michigan, Ann Arbor, MI 48109, USA; 3 Department of Chemistry and Program in Bioinformatics, Boston University, Boston, MA 02215, USA; 4 Departments of Chemistry, Physics, and Computer Science, University of Southern California, Los Angeles, CA 90089, USA

## Abstract

Many regulatory mechanisms require a high degree of specificity in protein-DNA binding. Nucleotide sequence does not provide an answer to the question of why a protein binds only to a small subset of the many putative binding sites in the genome that share the same core motif. Whereas higher-order effects, such as chromatin accessibility, cooperativity and cofactors, have been described, DNA shape recently gained attention as another feature that fine-tunes the DNA binding specificities of some transcription factor families. Our Genome Browser for DNA shape annotations (GBshape; freely available at http://rohslab.cmb.usc.edu/GBshape/) provides minor groove width, propeller twist, roll, helix twist and hydroxyl radical cleavage predictions for the entire genomes of 94 organisms. Additional genomes can easily be added using the GBshape framework. GBshape can be used to visualize DNA shape annotations qualitatively in a genome browser track format, and to download quantitative values of DNA shape features as a function of genomic position at nucleotide resolution. As biological applications, we illustrate the periodicity of DNA shape features that are present in nucleosome-occupied sequences from human, fly and worm, and we demonstrate structural similarities between transcription start sites in the genomes of four *Drosophila* species.

## INTRODUCTION

DNA shape analysis has been established in recent years as an approach that reveals determinants of protein-DNA binding specificity beyond the primary nucleotide sequence (1–4). Interactions between nucleotides within a binding site or its flanks are implicitly contained in the 3D structure of a DNA binding site. DNA shape is influenced by the core motif (5) and its flanking sequences (6) and therefore potentially characterizes binding sites with higher precision. In addition to taking into account interrelationships between nucleotide positions, DNA shape integrates over diverse nucleotide sequences that can give rise to similar DNA shapes, a phenomenon known as degeneracy of DNA sequence and structure. As a consequence, DNA shape was found to be evolutionarily conserved to a higher degree than is DNA sequence (7).

Based on these findings it seems advantageous to incorporate DNA shape features in motif scanning and *de novo* motif discovery methods (8–11). Another application for DNA shape analysis would be in the functional evaluation of genetic variation, which is commonly described in terms of nucleotide sequence (12,13). These and other applications will require the mapping of DNA shape features for entire genomes. To make the necessary data available we developed GBshape. Prediction of DNA shape features from nucleotide sequence is based on high-throughput methods for deriving DNA shape features, by using pentamers to mine results from all-atom Monte Carlo simulations of DNA fragments (14–16), and by predicting hydroxyl radical cleavage patterns based on an experimental dataset (17).

GBshape is a multi-species database currently containing whole-genome data for 94 organisms from groups of diverse species (Table 1). For each organism the database provides four genome browser tracks with annotations for Minor Groove Width (MGW), Propeller Twist (ProT), Roll and Helix Twist (HelT) (14). In a fifth track, GBshape shows hydroxyl radical cleavage annotations from the ·OH Radical Cleavage Intensity Database for double-stranded DNA (ORChID2) (18). These five DNA shape annotations were generated with the high-throughput prediction platform. DNA shape data can be visualized either qualitatively or downloaded as quantitative values via the GBshape user interface. GBshape contains DNA shape annotations for 91 genomes taken from the UCSC Genome Browser (19) and three additional genomes from plants, parasitic protists and bacteria (Table 1).

**Table 1.** Current number of genomes from diverse species in GBshape listed by UCSC Genome Browser organism group with additional groups added.

Organism group	Genome count
Mammals	47
Vertebrates	19
Deuterostomes	3
Insects	14
Nematodes	6
Fungi	1
Plants	1
Protists	1
Bacteria	1
Others	1
Total	94

We demonstrate the value of analyzing DNA shape annotations using GBshape by comparing the structural features of *in vivo* nucleosome binding sites from worm, fly and human (20) and the evolutionary conservation of DNA shape at transcription start sites (TSSs) across multiple *Drosophila* species (21). The GBshape database completes the family of DNA shape tools that includes DNAshape, a web server for high-throughput prediction of DNA shape features for up to 1 million base pairs (14), TFBSshape, a database of DNA shape features of transcription factor binding site motifs (22), and ORChID, a database and prediction tool for hydroxyl radical cleavage patterns (17,18).

## DATABASE

### Database architecture and methodology

**Figure 1.**
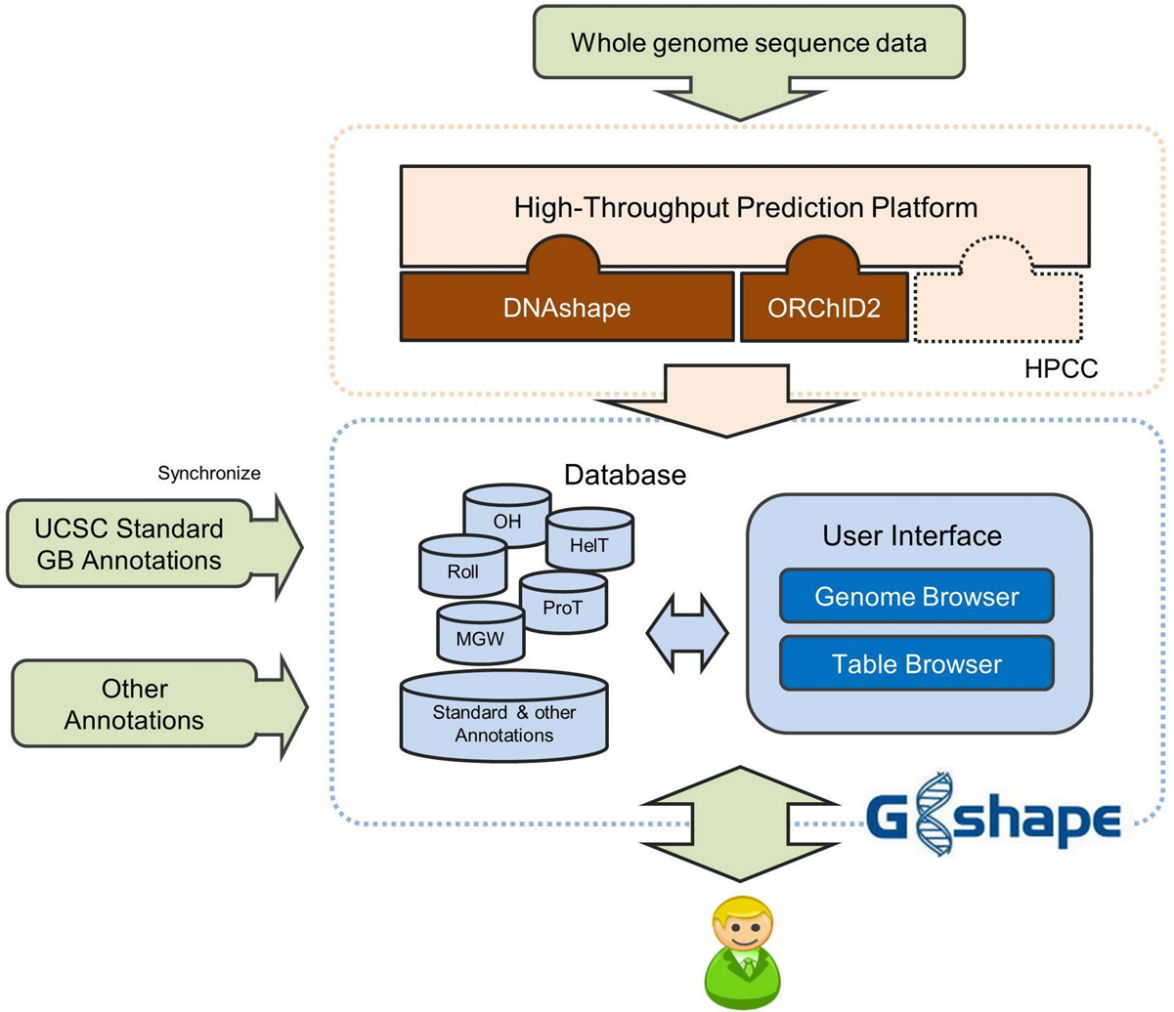
Architecture of the GBshape database. GBshape consists of a high-throughput prediction platform, data depositories and a user interface. DNA shape annotations of entire genomes can be generated by the high-throughput prediction platform, which runs on a high-performance computing cluster (HPCC), and, together with genome sequences and UCSC Genome Browser standard annotations, stored in the data depositories. The user interface provides multiple functionalities for users to either visualize or download structural annotations.

The core of our database is a high-throughput prediction platform (Figure 1) that we developed to generate DNA shape data for storage in GBshape. Whole genome sequence files (in FASTA format) for multiple species are subjected to the high-throughput prediction programs DNAshape (14) and ORChID2 (18) that are embedded in the GBshape platform. The GBshape prediction platform was designed to be extendable by plug-ins of other whole-genome annotation programs (Figure 1). The results of high-throughput prediction programs are converted to the bigWig data format, which can be displayed in a genome browser. The platform was developed in C++ and runs on a high-performance computing cluster (HPCC).

The GBshape database combines DNA shape data with standard UCSC Genome Browser annotations (19) and whole-genome sequence data. The sequences of 91 genomes (Supplementary Table S1) and corresponding standard annotations were downloaded from the UCSC Genome Browser (19). Although several genome assembly versions are available for many of these species, at this stage of development the most recent genome assembly for each species was chosen. The reference genome from *Saccharomyces cerevisiae* was identical with the one provided by the Saccharomyces Genome Database (23). Three additional reference genomes from *Arabidopsis thaliana* (24), *Plasmodium falciparum* (25) and *Escherichia coli* (26) that were not present in the UCSC Genome Browser were added to GBshape (Supplementary Table S1). The GBshape framework enables an easy expansion to additional genome assemblies, and users can submit a web form requesting the addition of specific genomes to our database. The GBshape database runs on MySQL (Figure 1).

The GBshape tracks for MGW, ProT, Roll and HelT were generated using our high-throughput method DNAshape (14). These DNA shape features were selected based on prior experimental studies demonstrating their important role in protein-DNA recognition, and include MGW (27–29), ProT (6), Roll (30) and HelT (28). Using pentamers as sliding windows, DNAshape mines all-atom Monte Carlo simulations (15,31) of 2121 DNA fragments of 10–27 bp in length. Each of the 512 unique pentamers is assigned the average value of all of its occurrences in the dataset at the central nucleotide for MGW and ProT and at the two central base pair (bp) steps of the pentamer for Roll and HelT. Each pentamer occurs on average 44 times in our Monte Carlo-generated dataset. The DNAshape method was validated against experimental data from X-ray crystallography, nuclear magnetic resonance spectroscopy and hydroxyl radical cleavage measurements (14).

We have shown that the hydroxyl radical, a small, uncharged, highly reactive molecule, reacts with the backbone of naked DNA in a manner that reflects the solvent accessible surface areas of the hydrogen atoms of the deoxyribose sugar, thus providing an experimental image of DNA backbone shape (18,32). To develop this chemical approach into a high-throughput method we performed hydroxyl radical cleavage experiments on 150 diverse DNA fragments of 40 bp in length. We devised a prediction algorithm, based on this database of experimental cleavage patterns, that uses a sliding tetramer window to predict the cleavage pattern for DNA sequences of any length (17). We subsequently extended this method by averaging the predicted cleavage patterns of both DNA strands to develop ORChID2, which we previously showed to be correlated with MGW and electrostatic potential (18). Thus, the ORChID2 pattern provides an experiment-based prediction of minor groove shape, which complements the Monte Carlo-based DNA shape features as an additional annotation track in GBshape.

### User interface

The GBshape user interface is a customized version of the UCSC Genome Browser that is hosted on our local server. The user interface contains some important functionalities of the UCSC Genome Browser, including the genome browser, table browser, the Basic Local Alignment Search Tool-like alignment tool (BLAT) and the ‘add custom tracks’ tool. The GBshape interface runs on a Linux-operated dual-core IBM server with Apache.

GBshape consists of two major tools—a genome browser and a table browser. The genome browser provides a graphical representation of DNA shape annotations along with standard genome browser annotations. The genome browser also supports text and sequence search functions to provide easy access to genomic regions of interest. The table browser enables data manipulation, downloads of multiple records and basic statistical analyses, which cannot be performed with the genome browser function.

**Figure 2.**
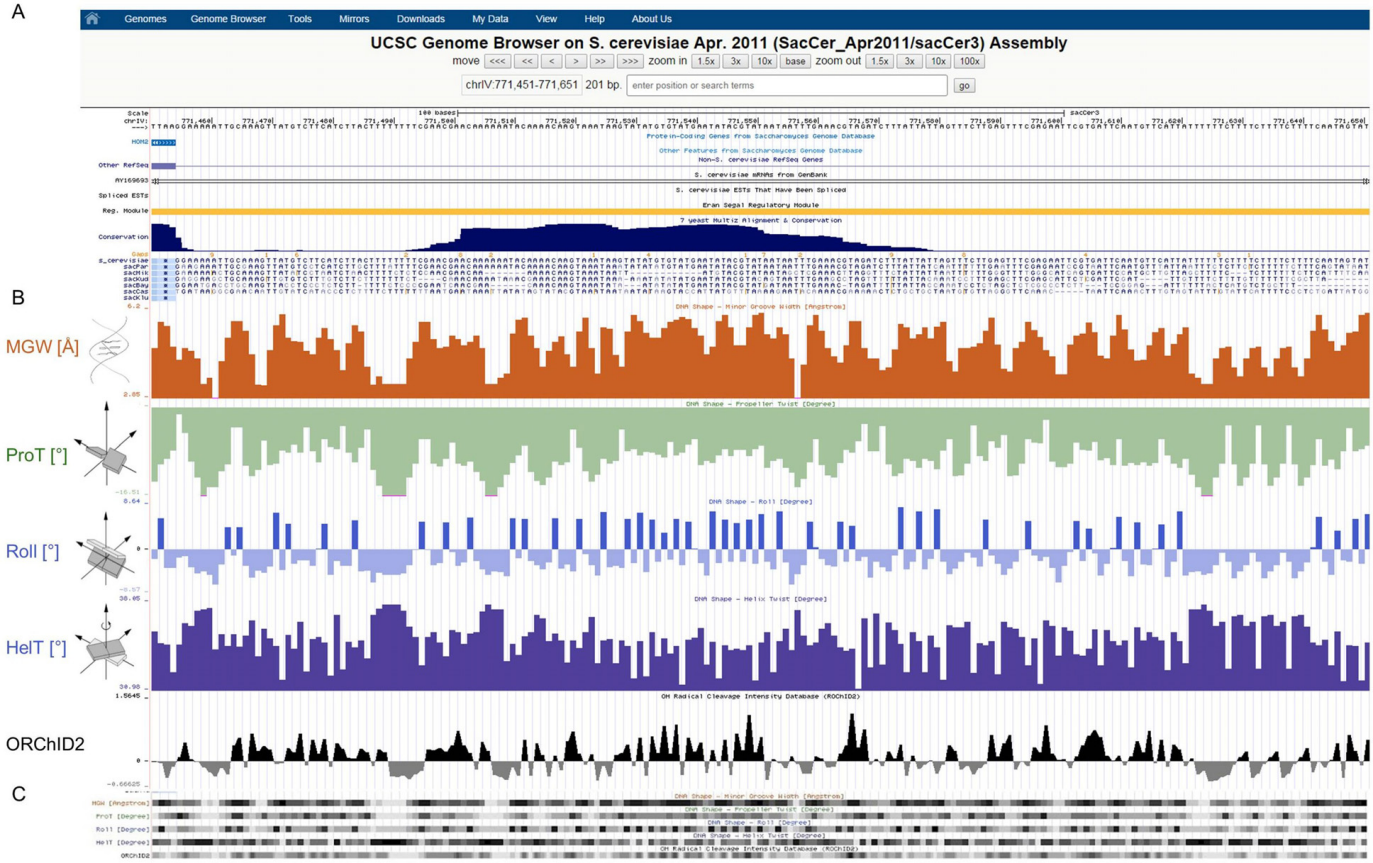
Visual display of GBshape annotations in the genome browser for a specific position in the *S. cerevisiae* genome. (A) Genome positions and UCSC Genome Browser standard annotation tracks. (B) DNA shape annotation tracks for MGW, ProT, Roll, HelT and hydroxyl radical cleavage intensity (ORChID2). (C) Heat map views for DNA shape annotations.

To visualize DNA shape annotations the user clicks on ‘Genome Browser’ in the navigation bar on the left of the GBshape homepage. On the Genome Browser Gateway page the user chooses an organism group, species genome, genome assembly, genome position and search terms of interest. After the ‘submit’ button is pressed, consolidated results for DNA shape annotations, together with standard genome annotations (Figure 2A), are shown on the display page. The shape annotations MGW, ProT, Roll, HelT and ORChID2 can be shown as quantitative plots (Figure 2B) or condensed into heat maps (Figure 2C).

The sequence-alignment tool, BLAT, can be used to search specific regions of the genome based on sequence similarity. To use BLAT, click on ‘Tools’ in the navigation bar at the top of the Genome Browser Gateway page, select ‘Blat’ in the pull-down menu, select a genome, assembly, query type, sort output and output type, and then press the ‘submit’ button. Genomic annotations can be viewed by clicking on the ‘browser’ link at the left of the search results. Supplementary Table S2 provides information on genomes for which BLAT supports a sequence search.

The view of the genome browser can be adjusted by using the buttons located near the top of the display page to move along the genome sequence, zoom in or zoom out, or by dragging and zooming the genomic position. The display type of an annotation track can be changed by selecting the pull-down menu from the track control panel at the bottom of the page. A heat map view can be shown for a track by setting the display type as ‘dense’ on the corresponding control panel. Users can upload their own tracks to compare with the existing annotations by using the function ‘add custom tracks’.

The table browser supports downloading and analysis of quantitative DNA shape annotations. To access these functions, click on ‘Table Browser’ in the navigation bar on the GBshape homepage. The Table Browser can also be found under the ‘Tools’ link in the navigation bar of the Genome Browser Gateway page. One can download DNA shape annotations for an entire genome or for a specified genomic region by setting parameters on the Table Browser page. Download of data for multiple regions is specified by setting ‘define regions’. Users can download data that match certain criteria by setting the ‘filter’ function, or manipulate data from different datasets by using the ‘intersection’ function. Output data can be exported in a variety of formats for further analysis or for use in other applications. Statistical correlations can be calculated over selected datasets, such as the correlation between data in different shape annotation tracks.

## BIOLOGICAL APPLICATIONS

### Nucleosome binding sites

Periodicity in nucleotide sequence has been detected in DNA sequences that wrap around histone octamers to form nucleosome core particles (33). The 10-bp periodicity of dinucleotide occurrence (34) and A-tract composition (1) mirrors the variation in width of the DNA minor groove as it is directed toward the histone core once every helical turn. We reported that the minor groove in nucleosome-bound DNA exhibits a 10-bp periodicity in MGW and electrostatic potential, and concluded that contacts of histone arginines with narrow minor groove regions are stabilized by the 10-bp shape-dependent periodicity in electrostatic potential (1).

A question that arises from these observations is whether periodic patterns in dinucleotide occurrence result in DNA shape features that guide nucleosome formation. Genome-wide nucleosome occupancy maps with thousands of nucleosome binding sites have been experimentally constructed by digesting intact chromatin with micrococcal nuclease followed by sequencing the underlying protected DNA fragments (MNase-seq) (34,35). We have used GBshape to infer structural features of these nucleosome-bound sequences.

We previously analyzed DNA shape features of 23 076 nucleosome-bound sequences from *Saccharomyces cerevisae* (34) and 25 654 from *Drosophila melanogaster* (35). We showed that analysis of shape profiles generated by the DNAshape and ORChID2 algorithms reveals a pronounced 10-bp periodicity in structural properties of nucleosomal DNA (14,18). The modENCODE consortium recently generated more extensive lists of nucleosome-bound sequences of much higher quality for human, *Drosophila melanogaster and Caenorhabditis elegans* (20).

**Figure 3.**
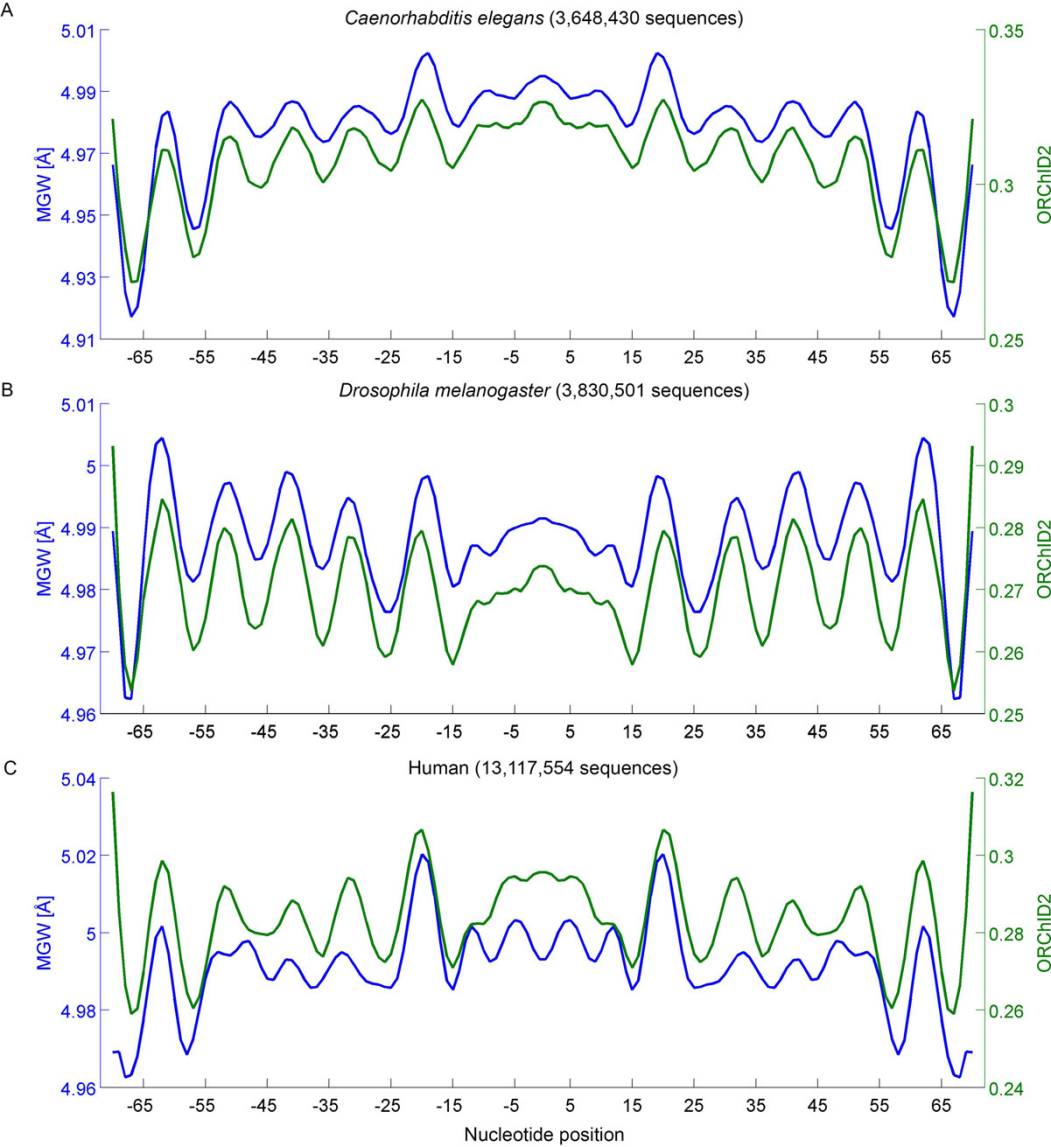
Variation in MGW (blue) and ORChID2 (green) signals on average in nucleosome sequences from the (A) *Caenorhabditis elegans*, (B) *Drosophila melanogaster* and (C) human genomes. Numbering of the nucleotide position starts with −1 and 1 for the central two base pairs, respectively.

**Figure 4.**
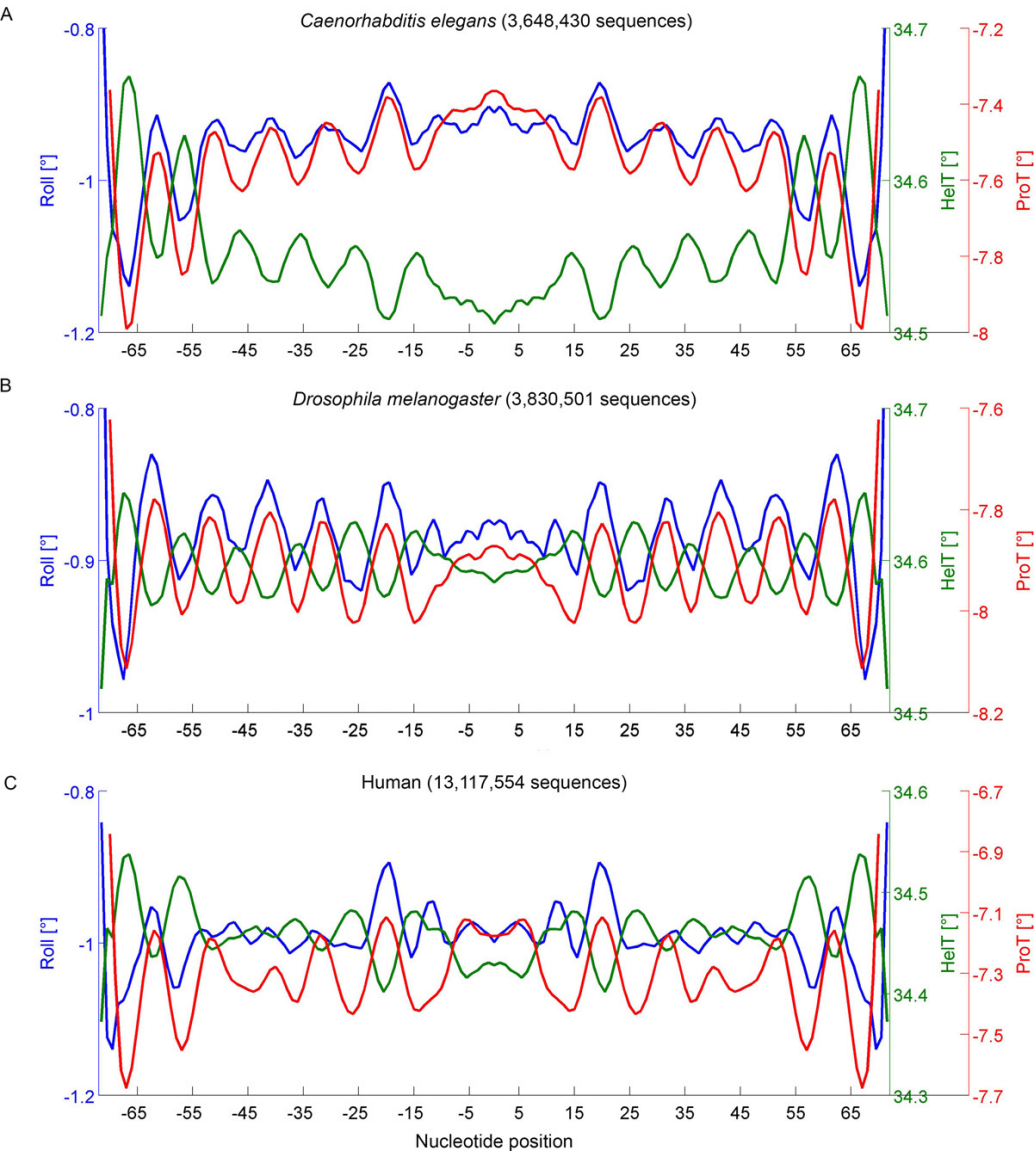
Variation in Roll (blue), HelT (green) and ProT (red) on average in nucleosome sequences from the (A) *Caenorhabditis elegans*, (B) *Drosophila melanogaster* and (C) human genomes. Numbering of the nucleotide position starts with −1 and 1 for the central two base pairs, respectively.

We have now used GBshape to predict MGW and compare this structural property to the ORChID2 pattern for these massive lists of 3.6 million from *Caenorhabditis elegans*, 3.8 million nucleosome-bound sequences from *Drosophila melanogaster* and 13.1 million from the human genome (Figure 3). The strong correlation between MGW and ORChID2 for all three organisms served as a validation of GBshape due to the independent approaches used to generate these predictions. Whereas the 10-bp periodicity was shared between human, fly and worm, details of the DNA shape profiles of nucleosomal DNA varied across species due to the different nucleotide compositions of these genomes. Analysis of the other DNA shape features Roll, HelT and ProT further confirmed the shared 10-bp periodicity as well as distinctions in DNA shape between nucleosome-bound sequences in these genomes (Figure 4). The maxima and minima of the MGW, Roll and ProT patterns overlapped, whereas the troughs in the HelT patterns matched the peaks in the other parameters, indicating a local helix unwinding at positions where a more positive Roll locally widens the minor groove.

### TSSs

TSSs are located at the 5′ end of genes where contacts with RNA polymerase II initiate transcription. A long-standing question in the field is how these positions can be identified in a genome using computational methods (36). Whereas the presence of conserved sequence elements, such as the TATA box and the Initiator (Inr) element, represent one possibility for identifying TSSs, nucleotide composition varies in Inr elements and in regions surrounding TSSs. Previous reports suggested that structural features, including DNA bending and melting, enhance protein binding at TSSs (36). We used GBshape as a high-throughput approach to annotate DNA shape features at TSSs of four different *Drosophila* species.

We derived data from paired-end cap analysis for gene expression experiments (21) to identify TSSs in the *D. melanogaster*, *D. simulans*, *D. sechellia* and *D. pseudoobscura* genomes. Transcription initiates from a range of positions at a given promoter, resulting in a frequency distribution that varies from ‘broad’ to ‘sharp’ between promoters (37). Depending on the analysis, a single representative TSS position for each promoter can be chosen based on the median position or the position with the maximum number of initiation events (peaks) within a given TSS distribution. For this analysis, we chose the peak position within each local frequency distribution in order to maximize the 5′ sequence alignments.

**Figure 5.**
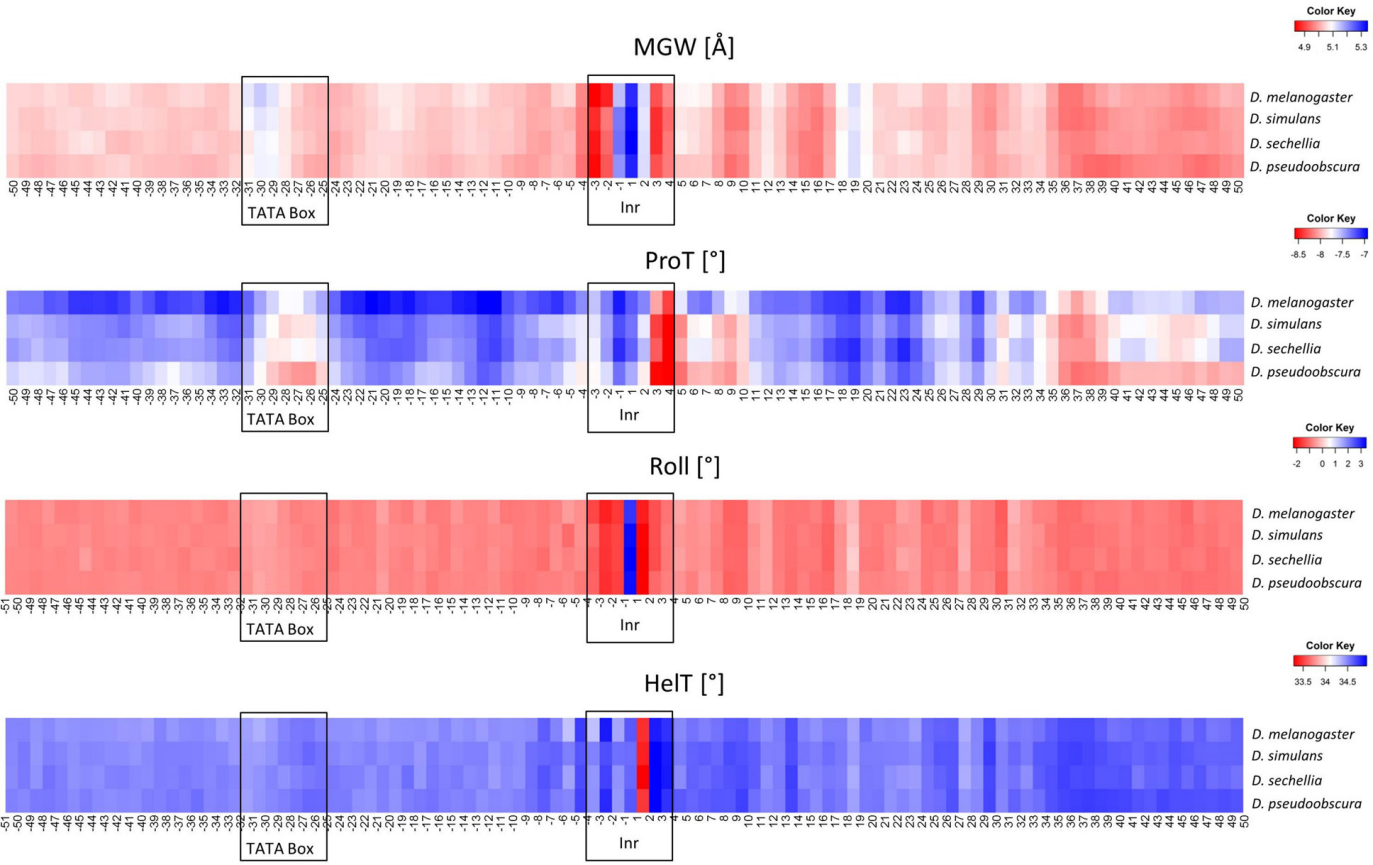
Average heat maps for four DNA shape features of TSSs and 50 bp up- and downstream in four fly species. The analysis is based on 3823 TSSs from the *D. melanogaster*, 6909 TSSs from the *D. simulans*, 7234 TSSs from the *D. sechellia* and 7397 TSSs from the *D. pseudoobscura* genomes. Column numbers in each heat map indicate the nucleotide position relative to the TSS. Black frames mark the locations of the Initiatior (Inr) element and TATA box.

Our DNA shape analysis of *Drosophila* TSSs revealed a clear structural signature for the Inr element despite the nucleotide sequence variation of this element. Moreover, specific DNA shape annotations of TSS regions were apparent for MGW, ProT, Roll and HelT (Figure 5). For each DNA shape feature the patterns were similar among the four *Drosophila* species, suggesting an evolutionary role of DNA structure. Whereas this effect merits further investigation, GBshape provides a platform that enables studies in which one can easily navigate between DNA sequence and shape information for a very large genomic datasets.

## CONCLUSIONS

We have developed a database of DNA shape annotations for whole genomes of 94 organisms. Given the emerging literature on the importance of DNA structural features in refining transcription factor binding specificities (2), this tool provides a framework for integrating DNA shape in whole-genome analyses. GBshape currently includes tracks for five structural features: MGW, ProT, Roll and HelT using DNAshape predictions (14), and hydroxyl radical cleavage intensity derived from ORChID2 (18). To demonstrate the utility of GBshape we analyzed structural features of nucleosome binding sites and TSSs. The availability of DNA shape annotations for entire genomes will enable the integration of DNA structure into genome analyses that currently are based only on nucleotide sequence.

## SUPPLEMENTARY DATA

Supplementary Data are available at NAR Online. See Supplementary Data for detailed author contributions.
